# Chemotherapy for advanced breast cancer: what influences oncologists' decision-making?

**DOI:** 10.1054/bjoc.2001.1733

**Published:** 2001-05

**Authors:** E A Grunfeld, A J Ramirez, E J Maher, D Peach, T Young, I P Albery, M A Richards

**Affiliations:** ICRF Psychosocial Oncology Group, GKT Medical School, St Thomas' Hospital, London SE1 7EH; Lynda Jackson Macmillan Centre, Mount Vernon Hospital, Northwood, Middlesex HA6 2RN

**Keywords:** palliative chemotherapy, advanced breast cancer, clinical decision-making

## Abstract

Chemotherapy is widely used in the management of patients with advanced breast cancer. However, a considerable proportion of patients experience toxic side effects without gaining benefit. This study aimed to elicit oncologists' views of the goals of chemotherapy for patients with advanced breast cancer and to elicit which factors are important in decisions to recommend chemotherapy to such patients. 30 oncologists underwent a semi-structured interview to examine their views of 5 goals of chemotherapy and of various disease, treatment and patient-related factors that might influence decisions to offer treatment. The clinicians also made decisions regarding treatment in relation to a hypothetical patient scenario under varying clinical conditions. Relief of symptoms and improvement of activity were rated as the most valuable and achievable goals of treatment. The patient's performance status, frailty and their wishes regarding treatment were the most important patient-related factors in determining decision-making. The most important disease/treatment-related factors were pace of the disease, previous poor response to chemotherapy, co-existing symptoms and concurrent medical conditions. The hypothetical scenario revealed that co-existing medical conditions, adverse previous response, increased age and depression would decrease the likelihood of recommending chemotherapy, whereas key symptoms (e.g. breathlessness) and the patient's goals would increase the likelihood. The findings suggest that British oncologists primarily aim to improve patients' physical function, although subjective factors, such as a patient's desire for anti-cancer treatment and their future goals, also influence decisions to offer treatment. © 2001 Cancer Research Campaign http://www.bjcancer.com


*British Journal of Cancer* (2001) **84**(9), 1172–1178
© 2001 Cancer Research Campaign
doi: 10.1054/ bjoc.2001.1733, available online at http://www.idealibrary.com on IDEAL®

### Chemotherapy for advanced breast cancer: what influences oncologists' decision-making?

**EA Grunfeld[1], AJ Ramirez[1], EJ Maher[2], D Peach[2], T Young[2], IP Albery[1] and MA Richards[1]**

[1]ICRF Psychosocial Oncology Group, GKT Medical School, St Thomas' Hospital, London SE1 7EH; [2]Lynda Jackson Macmillan Centre, Mount Vernon Hospital, Northwood, Middlesex HA6 2RN

**Summary** Chemotherapy is widely used in the management of patients with advanced breast cancer. However, a considerable proportion of patients experience toxic side effects without gaining benefit. This study aimed to elicit oncologists' views of the goals of chemotherapy for patients with advanced breast cancer and to elicit which factors are important in decisions to recommend chemotherapy to such patients. 30 oncologists underwent a semi-structured interview to examine their views of 5 goals of chemotherapy and of various disease, treatment and patient-related factors that might influence decisions to offer treatment. The clinicians also made decisions regarding treatment in relation to a hypothetical patient scenario under varying clinical conditions. Relief of symptoms and improvement of activity were rated as the most valuable and achievable goals of treatment. The patient's performance status, frailty and their wishes regarding treatment were the most important patient-related factors in determining decision-making. The most important disease/treatment-related factors were pace of the disease, previous poor response to chemotherapy, co-existing symptoms and concurrent medical conditions. The hypothetical scenario revealed that co-existing medical conditions, adverse previous response, increased age and depression would decrease the likelihood of recommending chemotherapy, whereas key symptoms (e.g. breathlessness) and the patient's goals would increase the likelihood. The findings suggest that British oncologists primarily aim to improve patients' physical function, although subjective factors, such as a patient's desire for anti-cancer treatment and their future goals, also influence decisions to offer treatment. © 2001 Cancer Research Campaign http://www.bjcancer.com

**Keywords**: palliative chemotherapy; advanced breast cancer; clinical decision-making

Chemotherapy is widely used in the management of advanced breast cancer, although there have been no randomized controlled trials to assess the benefits of chemotherapy as compared with best supportive care, in terms of survival or quality of life. Disease response, measured in terms of tumour shrinkage, is observed in between 40% and 60% of cases during controlled clinical trials (Benner et al, 1994; Leonard et al, 1995). This reduction in tumour bulk has been shown to be associated with relief of symptoms and improvement in quality of life (Baum et al, 1980). Furthermore, in individual cases, there is little doubt that chemotherapy prolongs survival by months or even years. If chemotherapy were without toxicity such benefits would be considered worthwhile even if only a small proportion of patients experienced them. The reality, however, is that a considerable proportion of patients experience toxicity without gaining benefit.

Despite the lack of evidence regarding the benefit of palliative chemotherapy in advanced breast cancer, there is some agreement among oncologists regarding the reasons for prescribing treatment. These goals include potential prolongation of life, the relief of cancer-related symptoms and the prevention of symptoms/complications associated with the disease (Rubens et al, 1992). Additionally, chemotherapy may be prescribed to maintain a patient's sense of hope, to reduce anxiety or because the patient expresses a wish to continue with treatment (Markman, 1997). In some cases chemotherapy is administered to avoid difficult communication situations (Porsoltz and Tannock, 1993).

Oncologists consider a range of factors when deciding whether or not to offer palliative chemotherapy, including objective disease and treatment-related factors and more subjective patient-related factors. Disease-related factors include sites of metastases and pace of disease, whereas treatment-related factors incorporate disease response to previous treatments and associated toxicity (Stoll, 1990). Patient-related factors influencing management choice include the patient's chronological and physical age, social support networks, and the interests of the patient's family (Stoll, 1990). In situations where the patient's prognosis is poor, and where less agreement exists among specialists regarding the appropriate course of action, the decision process will often be driven by these more subjective value judgements (Maher and Jeffries, 1990).

The extent to which different disease, treatment and patient-related factors predict the outcome of treatment with palliative chemotherapy for advanced breast cancer is largely unknown. Initial disease-free interval and abnormal liver function tests/liver metastases have been identified as important prognostic factors (Namer et al, 1990; Falkson et al, 1991; Gregory et al, 1993) but evidence relating to other disease and patient characteristics is conflicting. Preliminary work has been undertaken to identify the factors predicting patient-reported benefit from first-line palliative chemotherapy using a simple global measure of well-being (Ramirez et al, 1998). High levels of psychological distress and the presence of a dry mouth prior to treatment were found to predict feeling worse after treatment. These 2 factors as well as pre-treatment lack of energy, breathlessness and the presence of liver metastases predicted patients failing to complete treatment either because they died or stopped attending hospital.

*Received 13 September 2000*
*Revised 22 December 2000*
*Accepted 22 January 2001*

*Correspondence to:* EA Grunfeld

The paucity of evidence for survival or quality of life benefits of chemotherapy compared with supportive care and for factors which may predict benefit from palliative chemotherapy, means that oncologists offer palliative chemotherapy to women with advanced disease based on empirical grounds. In this context there is a lack of information regarding the factors influencing oncologists' decision-making in the management of advanced breast cancer. This study aimed to elicit oncologists' views of the goals of palliative chemotherapy and the relative importance assigned to tumour, treatment- and patient-related factors in decision-making regarding palliative chemotherapy for patients with advanced breast cancer.

Oncologists' views of the goals of chemotherapy were examined according to a model of decision-making. A theoretically driven approach was adopted in order to improve comparability across goals (the same questions were asked in relation to each goal) and also to explain why clinicians make certain decisions. The Subjective Expected Utility (SEU) theory of decision-making was selected (Edwards, 1954; Tversky, 1967). Using this approach 2 key factors believed to influence decision-making were examined. Firstly, does the clinician believe that each of the goals of chemotherapy is achievable (e.g. does the clinician believe that chemotherapy can prolong life)? Secondly, how valuable or important is that goal to the clinician (e.g. how worthwhile is it to prolong the patient's life)? This approach enabled the assessment of the relative priority of the separate goals when making decisions regarding chemotherapy. It also enabled the separate examination of the perceived effectiveness (expectancy) and perceived value (utility) associated with each of the goals. This theory has been applied previously to investigate health-related decision-making, including patient decisions regarding treatment for breast cancer (Stanton et al, 1998).

### PARTICIPANTS AND METHODS

All the specialist registrars and consultants in oncology working at two cancer centres were invited, by letter, to participate in the study. Of the 40 oncologists approached, 30(75%) agreed to participate. Those who declined did so either because they could not spare the time or because they were not involved in the treatment of patients with advanced breast cancer. All participating clinicians were currently involved in the treatment of women with advanced breast cancer. The participants comprised 20 specialist registrars (3 medical oncologists, 17 clinical oncologists) and 10 consultants (3 medical oncologists, 7 clinical oncologists). The median age of the clinicians was 35.5 years (range 30–53 years) and they had been practising in oncology for a median of 10.1 years (range 1–29 years). The sample contained 20 male and 10 female clinicians.

Clinicians who agreed to participate underwent a semi-structured interview lasting between 30–40 minutes. The interview schedule comprised 4 sections.

### Prescribing practice

The oncologists were asked to report their standard first-line chemotherapy (FLCT) and second-line chemotherapy (SLCT) regimens, the side effects associated with these chemotherapeutic agents and any circumstances under which they would not recommend chemotherapy due to potential side effects.

### The goals of palliative chemotherapy

Five key goals of palliative chemotherapy were identified through a literature search and by means of questioning relevant experts. These were prolongation of life, symptom relief, delaying the onset of symptoms, improving patient activity and maintaining the patient's hope of a positive outcome. The goals were examined within the framework of the SEU theory of decision-making. The perceived effectiveness of the goals of palliative chemotherapy comprised 2 questions. The clinicians were asked to rate on a 4-point scale (not at all, a little, moderately, very effective) how effective they thought chemotherapy would be in achieving each of the goals. They then estimated on a 5-point scale (none, a few, a moderate number, most, all) the proportion of patients that this goal could be achieved in. The mean of these 2 responses was taken as the perceived effectiveness (expectancy) score. The clinicians also rated (on a 10-point numerical scale) how valuable they perceived each of the goals to be with regard to a woman with advanced breast cancer. The clinician's perception of the efficacy of palliative chemotherapy may be influenced by the disease progression (i.e. chemotherapy regimens may be perceived to be less effective as the disease progresses) and therefore the same perceived effectiveness questions were asked for both first- and second-line chemotherapy. The value attached to each outcome would not be expected to change in relation to disease progression, as an outcome is either perceived as valuable or not. Therefore, value questions were asked only once and were phrased in relation to palliative chemotherapy.

Both the perceived effectiveness and value scores were log transformed so that each had a potential range of scores of 0 to 100. This was to ensure that the value scores (which had a higher possible maximum score due to the greater range of the rating scale for these questions) did not unduly influence the total SEU score. As the value and perceived effectiveness scores were rated on different scales it would not be appropriate to make a direct comparison between them. The SEU scores were calculated as: (perceived effectiveness score $\times$ value score)/100. This gave a potential range of 0 to 100.

### The importance of disease, treatment and patient factors in offering palliative chemotherapy

The importance of 10 disease- and treatment-related factors and 12 patient-related factors in decision-making regarding palliative chemotherapy was assessed. The influential factors were identified from a literature review and through discussion with relevant experts (oncologists, palliative care specialists, psychologists and a psychiatrist). Clinicians rated on a 4-point scale (not at all, a little, quite, very) the importance of each factor in a decision to give chemotherapy to a woman with advanced breast cancer for whom there were no hormonal treatments which would be of benefit. For the analysis this scale was collapsed to form 2 categories (1) not at all/a little, (2) quite/very. The number of clinicians rating each factor in the 2 categories was calculated. The data presented refer to the percentage of clinicians rating a particular factor as quite/very important in the decision.

### Scenario of decision-making for a hypothetical patient

The scenario examined which factors were important in the decision to recommend chemotherapy to a hypothetical patient with

© 2001 Cancer Research Campaign advanced breast cancer. This was felt to be more representative of the clinical situation than reference to the 'average patient'. Clinicians rated on a 5-point scale (definitely not, possibly, probably, almost definitely, definitely) how likely they would be to discuss and to recommend chemotherapy to a hypothetical patient under varying conditions (see Appendix).

The ratings were re-labelled 1 (definitely not) to 5 (definitely) for the analysis. The baseline score was defined as the rating obtained following the initial scenario. The scores obtained for each subsequent condition were then calculated and the difference between this score and baseline score was obtained. This difference score (for each of the 7 conditions) was plotted against the baseline score to ascertain the effect of each condition on the initial decision.

### Statistical analysis

Medians and inter-quartile ranges (IQR) are reported. Differences in the responses assigned to first- and to second-line chemotherapy were examined using the Wilcoxon Signed Ranks test. The Mann–Whitney U test was used to examine differences according to speciality, grading and gender.

### RESULTS

### Prescribing practice

The 3 most frequently reported standard first-line chemotherapy regimes were fluorouracil, epirubicin and cyclophosphamide (FEC) (reported by 59% of clinicians), cyclophosphamide, methotrexate and fluorouacil (CMF) (31%) and mitozantrone, mitomycin and methotrexate (MMM) (24%). For second-line chemotherapy the most frequently cited standard regime was paclitaxel (Taxol) (cited by 41% of clinicians), followed by MMM (28%) and vinorelbine (21%). The most frequently reported side effects for both first- (FLCT) and second-line chemotherapy (SLCT) were alopecia (89% FLCT, 86% SLCT), nausea and vomiting (83% FLCT, 72% SLCT), fatigue (66% FLCT, 45% SLCT) and neutropenia (48% FLCT, 44% SLCT). The patient's preferences, performance status and frailty were the commonest reasons for not prescribing chemotherapy due to potential side effects.

The estimated extra time a patient might expect with FLCT (mean 4.9 months, SD 2.7) was significantly greater than the 2.9 months (SD 1.7) estimated for SLCT ($Z = -4.43$, $P < 0.05$). The medical oncologists were more optimistic regarding the extra time afforded by FLCT (5.6 months, SD 2.0) than were the clinical oncologists (4.3 months, SD 2.8) ($Z = -2.04$, $P < 0.05$). The 2 specialities were not significantly different in their estimates for SLCT ($Z = -1.50$, $P = 0.14$). There were no significant differences between the genders or grades (specialist registrar or consultant) with regard to survival estimates.

### Goals of palliative chemotherapy

#### *Perceived effectiveness of the goals of palliative chemotherapy*

The most achievable goals in relation to FLCT (see Table 1) were viewed equally to be symptom relief, maintenance of hope and improvement of activity (with a median rating of 57.1). The overall ranking of the goals for SLCT was similar to that of FLCT, except that improvement of activity received a lower comparative rating. The perceived effectiveness scores for each of the 5 goals assigned for SLCT were significantly lower than for first-line chemotherapy (all $P$ values < 0.01).

#### *Value of palliative chemotherapy*

The value questions were phrased in relation to palliative chemotherapy and did not examine FLCT and SLCT separately. The value scores attached to each of the goals of chemotherapy differed somewhat from the respective perceived effectiveness scores (Table 1). Improvement of activity was rated as the most valued goal of chemotherapy (median score 98.2) followed by symptom relief (median score 70.3). However, maintenance of hope was rated as the fourth most valuable use of palliative chemotherapy, whereas it had been ranked as the most achievable goal for both first- and second-line chemotherapy.

Although prolongation of life was only rated as the third most valued goal of palliative chemotherapy further examination of the data revealed that the value ratings were dependent upon of the amount of extra time a woman could expect. Clinicians were asked to indicate how valuable they believed it would be for a patient with advanced breast cancer to have an extension of life of between 1 and 24 months. The results are represented in Figure 1. It can be seen that an extension of life of 12 months or more was valued most highly and that there was greater agreement (less variability) among the clinicians regarding survival times of 18 and 24 months. A similar time-dependent pattern was apparent for questions relating to delaying the onset of symptoms although the clinicians continued to vary in their value ratings even with increasing delay times (Figure 2). There were no significant differences in the value scores assigned by the oncologists according to speciality, gender or grading.

**Table 1** The median effectiveness (expectancy) and value (utility) scores assigned to each of the potential goals of chemotherapy. The inter-quartile ranges are shown in parentheses

| Goal | Median effectiveness score | | Median value score |
|---|---|---|---|
| | FLCT | SLCT | |
| Prolongation of life | 42.9 (28.6) | 28.6 (3.6) | 68.2 (11.9) |
| Symptom relief | 57.1 (14.3) | 35.7 (28.6) | 70.3 (15.2) |
| Delaying onset of symptoms | 46.4 (28.6) | 0 (8.6) | 53.3 (18.9) |
| Improvement of activity | 57.1 (16.1) | 28.6 (14.3) | 98.2 (14.8) |
| Maintaining hope | 57.1 (14.3) | 42.9 (28.6) | 66.7 (25.0) |

 *© 2001 Cancer Research Campaign*

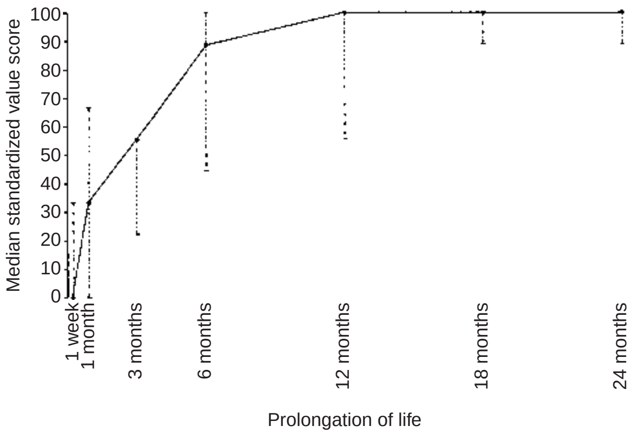

**Figure 1** The median standardized value scores assigned to various periods by which life could be extended (for a woman with metastatic breast cancer). The dashed lines represent the total range of responses assigned to each time period

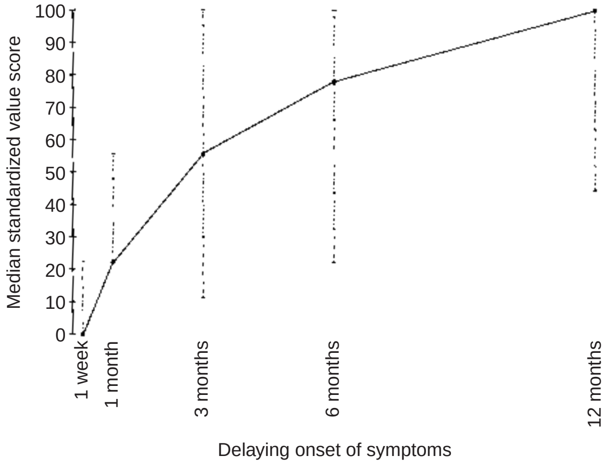

**Figure 2** The median standardized value scores assigned to various periods by the onset of symptoms could be delayed (for a woman with metastatic breast cancer). The dashed lines represent the total range of responses assigned to each time period

### *Subjective expected utility (SEU) scores*

The SEU scores were calculated as the perceived effectiveness score (for FLCT or SLCT) multiplied by the value score divided by 100 (see Table 1 for the scores for the individual scales). The median SEU scores for FLCT and SLCT are shown in Figure 3. The highest SEU score in relation to FLCT was obtained for improvement of activity (median 48.7, IQR 21.7). The median score for symptom relief was slightly lower although there was less variability in the rating of this goal (median 44.4, IQR 11.4). Maintenance of hope was assigned the third highest SEU score (median 39.7, IQR 19.9), followed by prolongation of life (median 25.2, IQR 13.1) and finally, delaying the onset of symptoms (median 21.0, IQR 18.1).

For SLCT the highest SEU score was obtained for maintenance of hope (median 31.8, IQR 19.8) followed closely by improvement of activity (median 28.6, IQR 15.3) and symptom relief (median 25.4, IQR 18.3). The 2 remaining goals were rated in the same order as for FLCT; prolongation of life (median 17.7, IQR 8.6) and delaying the onset of symptoms (median 0.0, IQR 12.7). SEU scores for SLCT were significantly lower than for FLCT (Figure 3);

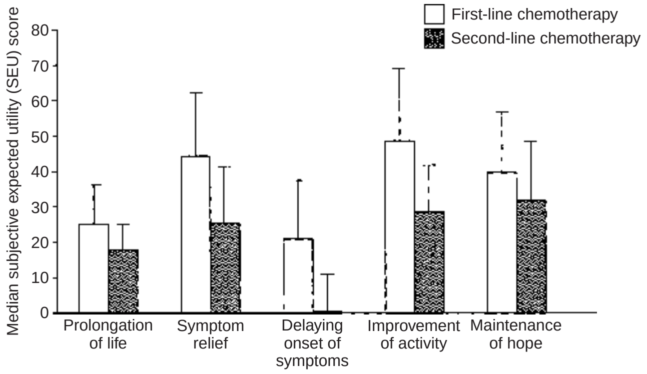

**Figure 3** The median subjective expected utility (SEU) scores for each goal of palliative chemotherapy presented according to first- and second-line chemotherapy. The error bars represent the inter-quartile range prolongation of life (Z = –3.91, $P < 0.01$), symptom relief (Z = – 4.26, $P < 0.01$), delaying symptoms (Z = – 4.20, $P < 0.01$), improving activity (Z = –3.65, $P < 0.01$) and maintenance of hope (Z = –3.77, $P < 0.01$).

The only significant difference to emerge between the SEU scores obtained from the clinical and medical oncologists was for symptom relief in relation to FLCT. Clinical oncologists produced significantly lower SEU scores for symptom relief than medical oncologists (Z = –2.15, $P < 0.05$). There were no significant differences in the SEU scores according to oncologist's grade or gender.

### Factors of importance in decision making regarding palliative chemotherapy

Three patient-related factors were most frequently reported to affect the decision to recommend palliative chemotherapy (Table 2). These were the patient's current performance status, the patient's wish to receive/not receive chemotherapy and the frailty of the patient. The disease/treatment-related factors rated as important were the pace of the disease, previous response to chemotherapy and the toxicity experienced, the presence of other symptoms/medical problems and the site of the metastases. The factors rated as least important in the decision to offer treatment were language barriers, the patient's education level and their access to transportation.

A comparison of the responses of medical and clinical oncologists revealed that significantly more clinical oncologists rated the site of the metastases (Z = – 0.716, $P = 0.47$) and the patient's previous response to chemotherapy (Z = – 0.694, $P = 0.49$) as important in the decision. There were no significant differences between the responses according to the clinician's gender or grading.

### Scenario of decision-making for a hypothetical patient

Following the initial scenario 65% of clinicians indicated that they would almost definitely or definitely *discuss* chemotherapy with the patient. However, only 20% indicated they would actually *recommend* chemotherapy to the patient. Several conditions were found to differ significantly from the baseline measurement (using the Wilcoxon Signed Ranks Test) suggesting that each might influence the original decision. Breathlessness (Z = –3.91, $P < 0.05$) was found to increase the likelihood of the oncologist recommending treatment. An increase in age of 20 years (Z = –2.45, $P < 0.01$), concurrent illnesses (Z = –2.97, $P < 0.05$), adverse

© 2001 Cancer Research Campaign

**Table 2** The percentage of clinicians rating each factor as quite or very important in the decision to give palliative chemotherapy to a woman with metastatic breast cancer. The factors are grouped according to patient and tumour-related factors

| Tumour/treatment-related factors | % of responses | Patient-related factors | % of responses |
|---|---|---|---|
| Pace of disease | 89.7 | Performance status | 96.6 |
| Previous response to chemotherapy | 86.2 | Patient's wishes | 96.6 |
| Symptoms other than pain | 86.2 | Frailty | 93.1 |
| Concurrent medical conditions | 82.8 | Age | 58.6 |
| Site of metastases | 79.3 | Social support | 51.7 |
| Toxicity with previous chemotherapy | 79.3 | Anxiety | 44.8 |
| Pain | 55.2 | Depression | 44.8 |
| Previous response to hormone therapy | 37.9 | Patient's family's wishes | 37.9 |
| ER/PR status | 27.6 | Pre-morbid personality | 27.6 |
| Histological type/grade | 24.1 | Language barriers | 20.7 |
| | | Education level | 13.8 |
| | | Access to transportation | 10.3 |

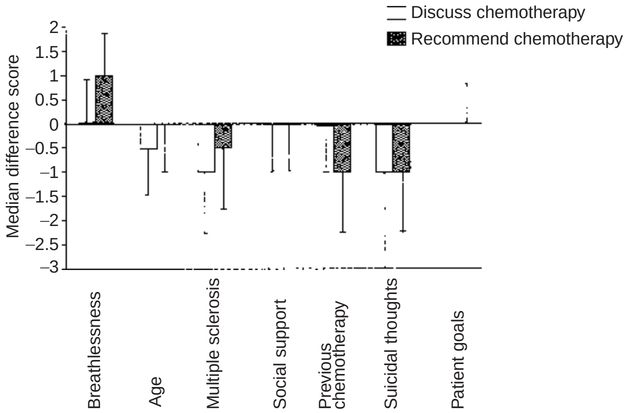

**Figure 4** The baseline scores for the discussion and recommendation of chemotherapy were taken as the responses following the initial scenario (possible range 1–5). The clinicians' responses for each of the subsequent conditions (possible range 1–5) was subtracted from their baseline score to obtain a difference score. The baseline score obtained is represented as zero on the graph. The median difference score obtain for each condition was then plotted against this zero axis. A positive number indicates an increased likelihood of discussing or recommending chemotherapy to the patient. A negative number indicates a reduced likelihood of discussing or recommending chemotherapy. The error bars represent the inter-quartile range previous experience of chemotherapy ($Z = –3.30$, $P < 0.05$) and depression ($Z = –3.54$, $P < 0.01$) were shown to decrease the likelihood of the oncologist recommending chemotherapy (Figure 4).

### DISCUSSION

There was a stronger belief among oncologists regarding the effectiveness of FLCT and this reflects the greater response rates published for FLCT rather than SLCT (Gregory et al, 1993; Benner et al, 1994). The estimates of extra survival time provided by the oncologists in this study are comparable with the 4 to 6 months for FLCT and 2 to 3 months for SLCT reported in the literature (Benner et al, 1994; Leonard et al, 1995).

### The goals of palliative chemotherapy

Both improvement of activity and symptom relief were rated as the most valuable and achievable goals of palliative chemotherapy. Improvement in symptoms is often associated with an observable improvement in the patient's physical functioning (Dodwell et al, 1993) which consequently may enable patients to carry out their daily activities, an outcome highly valued by both patients and clinicians (Sutherland et al, 1990; Dodwell et al, 1993). An objective response to chemotherapy (measured as a reduction in tumour bulk) is associated with improvement in both symptoms and performance status (Baum et al, 1980; Ramirez et al, 1998; Geels et al, 2000) and therefore clinicians would perceive these 2 goals as achievable.

This study did not examine the influence of individual chemotherapeutic drugs on evaluations of the goals of palliative chemotherapy. It is probable that clinicians would consider whether the likely response (i.e. a reduction in symptoms) would be great enough to offset potential toxic effects. This discussion may be particular relevant to the use of taxanes as SLCT agents, as certain agents (i.e. docetaxel) are known to be associated with serious side effects such as oedema with or without pleural effusion (Vogel and Nabholtz, 1999). However, it is known that cancer patients are prepared to undergo radical treatment even if this is only associated with a potentially small reduction in symptoms or minimal prolongation of life (Slevin et al, 1990). Currently a study is being undertaking to examine both oncologists' decision-making and patients' perceptions of treatment within clinical situations and therefore information regarding actual clinical decisions should be available at the end of the study.

The clinicians reported that it was possible to maintain a patient's sense of hope through the prescription of chemotherapy. Despite the low-value score assigned to this goal the high SEU score suggested that it would probably be one of the main reasons for prescribing second-line chemotherapy. Previous research has suggested that European oncologists are less likely than their American counterparts to perceive maintenance of hope as an important part of palliative treatments (Maher et al, 1992) and our results suggest that for first-line chemotherapy this may be so.

### Factors influencing decisions regarding palliative chemotherapy

Poor performance status and frailty were rated as very important in decisions to recommend chemotherapy, both of which have previously been identified as indicators of poor outcome from

 *© 2001 Cancer Research Campaign*

chemotherapy (Rubens et al, 1992). This study's identification of several primary patient-related factors is not in agreement with previous studies that have highlighted the importance of tumour/treatment factors (Derber and Thompson, 1990; McGuire et al, 1991). This may, however, reflect the different methodologies employed; the current study presented both disease- and patient-related factors whereas previous studies have placed greater reliance on tumour/treatment factors as this information is routinely requested by clinicians.

Within the decision-making scenarios, being older, having a concurrent medical condition, having had a difficult previous experience of chemotherapy, being depressed and the patient's future plans were all found to decrease the likelihood of clinicians recommending chemotherapy. All these factors affect patients' tolerance to treatment and possibly the outcome of treatment (Rubens et al, 1992). Breathlessness, however, was shown to increase the likelihood of the oncologist recommending chemotherapy. This factor relates to the goal of symptom relief which was viewed as an achievable goal of chemotherapy by the clinicians.

The present study elicited important information regarding oncologists' perceptions of the goals of palliative chemotherapy and the factors they consider when making decisions regarding the treatment of patients with advanced breast cancer. The SEU model provided a good starting point from which to investigate oncologists' treatment-based decision-making regarding women with advanced breast cancer. By examining expectancy (perceived effectiveness) and value separately it has allowed us to demonstrate that even though some of the goals (e.g. maintenance of hope) are not highly valued by oncologists, these goals may be perceived as easy to achieve and therefore chemotherapy may be prescribed for this purpose. The study, however, relied on generalized questions and hypothetical scenarios to elicit this information and therefore responses may not reflect actual clinical practice. This may also explain why few differences were found between the different clinical specialities and grades. A study is currently being undertaken to examine oncologists' decision-making with individual patients in clinical situations.

### ACKNOWLEDGEMENTS

This study formed part of a larger programme of research commissioned by the NHS R&D Cancer Programme (Grant NCP/G01). The authors would also like to express their gratitude to the consultants and specialist registrars who took part in this study.

### REFERENCES

Baum M, Priestman T, West R and Jones E (1980) A comparison of subjective responses in a trial comparing endocrine with cytotoxic treatment in advanced carcinoma of the breast. In: *Proceedings of the Second EORTC Breast Cancer Working Conference*. pp 223–226 Pergamon Press: Oxford

Benner SE, Fetting JH and Brenner MH (1994) A stopping rule for standard chemotherapy for metastatic breast cancer: lessons from a survey of Maryland oncologists. *Cancer Invest* **12**(5): 451–455

Derber RB and Thompson GG (1990) Variations in breast cancer: lessons treatment decisions and their impact in mounting trials. *Control Clinical Trials* **11**(5): 353–373

Dodwell DJ, Rathmell AJ and Ash DV (1993) Assessment of palliative chemotherapy: a step beyond response. *Clin Oncol* **5**: 114–117

Edwards W (1954) The Theory of Decision Making. *Psychol Bull* **51**: 380–417

Falkson G, Gelman R, Ralkson CI, Glick J and Harris J (1991) Factors predicting response, time to treatment failure, and survival in women with metastatic breast cancer treated with DAVTH: a prospective eastern co-operative oncology group study. *J Clin Oncol* **9**: 2153–2161

Geels P, Eisenhauer E, Bezjak A, Zee B and Day A (2000) Palliative chemotherapy: objective tumour response is associated with symptom improvement in patients with metastatic breast cancer. *J Clin Oncol* **18**: 2395–2405

Gregory WM, Smith P, Richards MA, Twelves CJ, Knight RK and Rubens RD (1993) Chemotherapy of advanced cancer: outcome and prognostic factors. *British Journal of Cancer* **68**: 988–995

Leonard RCF, Rodger A and Dixon JM (1995) Metastatic breast cancer. In Dixon JM (Ed) *ABC of Breast Diseases*. pp 45–48 BMJ Publishing Group: London

Maher EJ and Jefferis AF (1990) Decision making in advanced cancer of the head and neck: variation in the views of medical specialists. *Journal of the Royal Society of Medicine* **83**: 356–359

Maher EJ, Coia L, Duncan G and Lawton PA (1992) Treatment strategies in advanced and metastatic cancer: differences in attitude between the USA, Canada and Europe. *Int J Radiat Oncol Phys* **23**(1): 239–244

Markman M (1997) Salvage chemotherapy of malignant disease: importance of precisely defining goals of treatment. *Cancer Res Clin Oncol* **123**: 467–468

McGuire WL, Tandon AK, Allred DC, Chamness GC, Ravdin PM and Clark GM (1991) Prognosis and treatment decisions in patients with breast cancer without axillary node involvement. *Cancer* **70**(6): 1775–1781

Namer M, Mercier M, Hurteloup P, Bonneterre J and Bastit P (1990) Prognostic factors of metastasised breast cancer patients. *Breast Cancer Res and Treat* **16**: 60

Porzsolt F and Tannock I (1993) Goals of palliative cancer therapy. *J Clin Oncol* **11**(2): 378–381

Ramirez AJ, Towlson KE, Leaning MS, Richards MA and Rubens RD (1998) Do patients with advanced breast cancer benefit from chemotherapy? *British Journal of Cancer* **78**(11): 1488–1494

Rubens RD, Towlson KE, Ramirez AJ, Coltart S, Slevin ML, Terrell C and Timothy AR (1992) Appropriate chemotherapy for palliating advanced cancer. *BMJ* **304**: 35–40

Slevin ML, Stubbs L, Plant HJ, Wilson P, Gregory WM, Armes PJ and Downer SM (1990) Attitudes to chemotherapy: comparing views of patients with cancer with those of doctors, nurses and general public. *BMJ* **300**: 1458–1460

Stanton AL, Stanton AL, Estes MA, Estes NC, Cameron CL, Danoff-Burg S and Irving LM (1998) Treatment decision making and adjustment to breast cancer: a longitudinal study. *Journal of Consulting and Clinical Psychology* **66**(2): 313–322

Stoll BA (1990) Choosing between cancer patients. *Journal of Medical Ethics* **16**: 71–74

Sutherland HJ, Lockwood GA and Boyd NF (1990) Ratings of the importance of quality of life variables: therapeutic implications for patients with metastatic breast cancer. *J Clin Epidemiol* **43**(7): 661–666

Tversky A (1967) Additivity, utility and subjective probability. *Journal of Mathematical Psychology* **4**: 175–201

*© 2001 Cancer Research Campaign* 

### APPENDIX

### Scenario of decision-making for a hypothetical patient

A 48-year-old woman was initially diagnosed at another hospital with a 3 cm, 2 lymph node positive, grade III ductal carcinoma 4 years ago. She underwent breast conserving therapy including tumorectomy, axillary clearance and radiotherapy to the breast. She then received 6 cycles of standard adjuvant CMF with tamoxifen. Her periods stopped during chemotherapy. Recurrence was first detected in the supraclavicular fossa 6 months ago. She was treated with radiotherapy and her hormonal treatment was changed to anastrozole (Arimidex). She recently had a chest infection that has now cleared. However, a chest X-ray was undertaken which shows small pulmonary nodules highly suggestive of metastatic disease. She is said to be asymptomatic. She is now being referred to you for further management. You have never met the patient, but are aware of this information prior to the consultation.

The following conditions are then introduced. After each condition the clinician was asked to refer back to the original scenario (so the conditions were not additive).

1. You now learn that the patient is breathless.
2. The woman is 68 years old, rather than 48 years old.
3. When you meet the patient, she is in a wheel chair and tells you that she has been house-bound by multiple sclerosis for the past 10 years.
4. The patient tells you that she lives alone and has no support at all.
5. The patient tells you that the adjuvant CMF was the worst experience of her life.
6. The patient tells you that she is feeling low, almost to the point of wanting to end her life.
7. The patient tells you that she is determined to be well enough to visit her daughter in Canada for Christmas in 6 months time.

 © 2001 Cancer Research Campaign
